# Supplementary material for: RNA:DNA hybrids in the human genome have distinctive nucleotide characteristics, chromatin composition, and transcriptional relationships
Source: Epigenetics Chromatin. 2015 Nov 16;8:46. doi: 10.1186/s13072-015-0040-6 (PMC4647656; doi:10.1186/s13072-015-0040-6)
Supplement: Supplementary file 1 — 10.1186/s13072-015-0040-6 Supplemental Results and Methods. [file 13072_2015_40_MOESM1_ESM.pdf]

RNA:DNA hybrids in the human genome have distinctive nucleotide characteristics, chromatin composition, and transcriptional relationships.

Julie Nadel, Rodoniki Athanasiadou, Christophe Lemetre, N. Ari Wijetunga, Pilib Ó Broin, Hanae Sato, Zhengdong Zhang, Jeffrey Jeddeloh, Cristina Montagna, Aaron Golden, Cathal Seoighe, John M. Greally

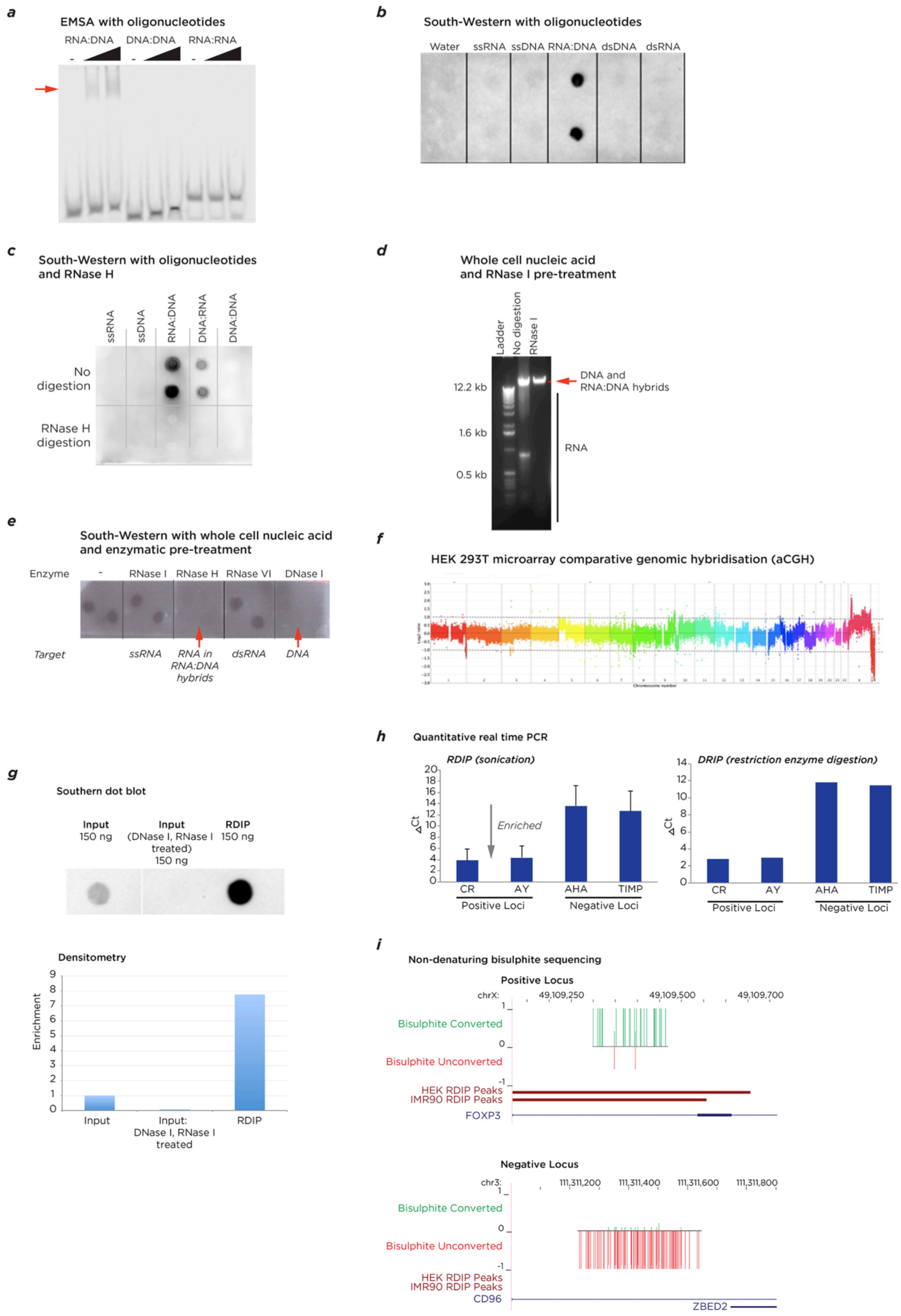

Figure S1: RDIP validation studies.

(a) An electrophoretic mobility shift assay (EMSA) showing the S9.6 antibody to bind only to oligonucleotides annealed as RNA:DNA hybrids, not dsDNA or dsRNA.

(b) A South-Western blot showing the S9.6 antibody to bind only to RNA:DNA hybrids, not dsDNA, dsRNA, ssDNA or ssRNA.

(c) The same as (b) but with pre-treatment with RNase H, which digests RNA when in an RNA:DNA hybrid, showing that the signal from South-Western blotting is eliminated by this pre-treatment.

(d) Gel electrophoresis showing whole cell nucleic acid and the effects of RNase I pre-treatment.

(e) A South-Western blot showing the effects on whole cell nucleic acid of pre-digestion with RNase I (to remove ssRNA), RNase H, RNase VI (to remove dsRNA) and DNase I (to remove DNA). As expected, both RNase H and DNase I eliminate the signal from the S9.6 antibody, consistent with the antibody detecting RNA:DNA hybrids specifically.

(f) Cloning of four HEK 293T cell lines allowed us to select one with the least amount of chromosomal rearrangement, as shown by the microarray comparative genomic hybridization shown.

(g) Following immunoprecipitation using S9.6, we performed a dot blot with the TERRA telomeric RNA as a probe. Equivalent amounts of input and immunoprecipitated material showed a >7x enrichment of TERRA signal, with the expected elimination of the majority of signal with pre-treatment using DNase I and RNase I (testing for non-specific background).

(h) The immunoprecipitated material was tested at candidate peaks (CR and AY loci) compared with loci not found to form peaks (AHA, TIMP) using quantitative real time PCR. The y axis shows the lower cycle number used to amplify immunoprecipitated material at the positive loci, with ~28 (=256) fold enrichment observed. On the left is shown the enrichment following the nucleic acid sonication approach of RDIP, on the right the results of restriction enzyme pre-digestion of DRIP.

(i) In the upper part of the panel, a locus with peaks called in both IMR-90 and HEK 293T cells is shown with the results of non-denaturing bisulphite sequencing revealing substantial conversion, indicating single strandedness of the DNA locally, consistent with the presence of an RNA:DNA hybrid, whereas the lower part of the panel shows no such conversion at a locus where no peak was called in either cell type.

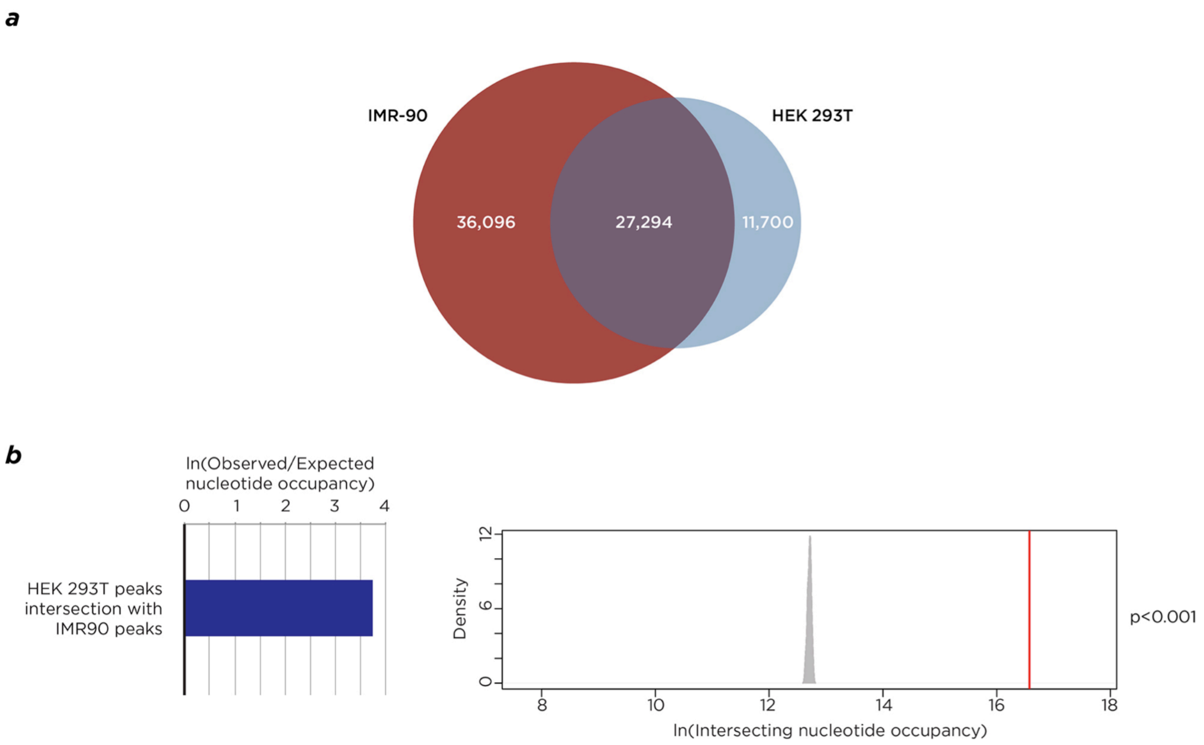

Figure S2: Concordance of RNA:DNA hybrid peaks in IMR-90 and HEK 293T cells.

The RDIP-seq assay identified tens of thousands of RNA:DNA hybrid peaks in each of the two cell types tested (a). The degree of overlap was tested using permutation analysis, with a significant enrichment for overlap shown in (b).

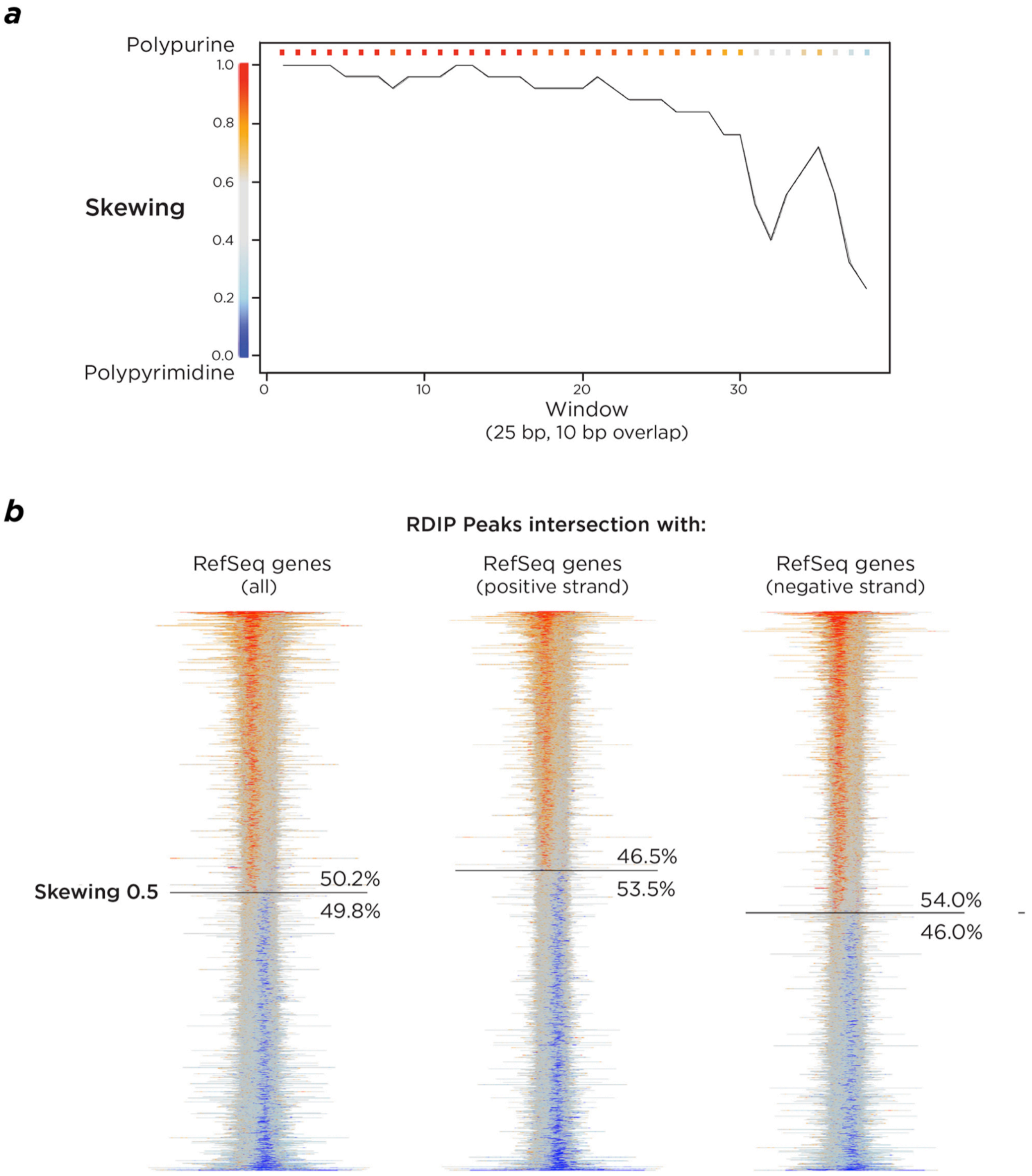

Figure S3: Visualizing the polypurine:polypyrimidine skewing in RNA:DNA hybrids.

Panel (a) shows how we depict skewing in each peak using red to indicate polypurine skewing and blue polypyrimidine skewing. We sort the peaks by degree of skewing overall in (b). We find that the polypurine enrichment is displaced to the left and polypyrimidine to the right of peaks, which is consistent with our library preparation technique (discussed in the main text). We also find that there is a small bias against polypurine enrichment being present in the same transcriptional orientation as the RefSeq gene.

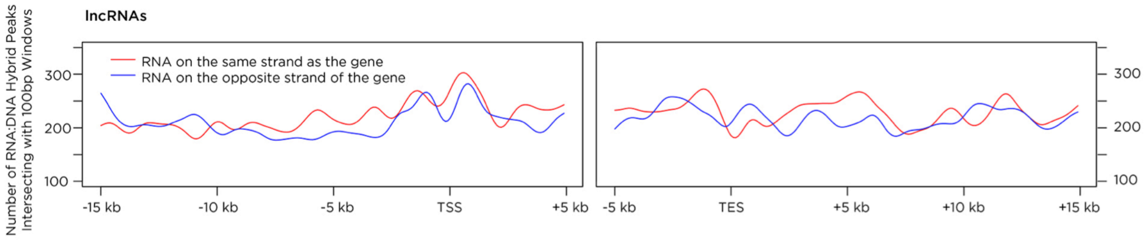

Figure S4: Metaplot of RNA:DNA hybrids in long non-coding RNAs (lncRNAs).

We show the RNA:DNA hybrid distributions on lncRNAs, those with the RNA on the transcribed strand in red, with blue showing the frequency on the non-transcribed stand. A modest enrichment for both strands is seen in the region near the transcriptional start site (TSS).

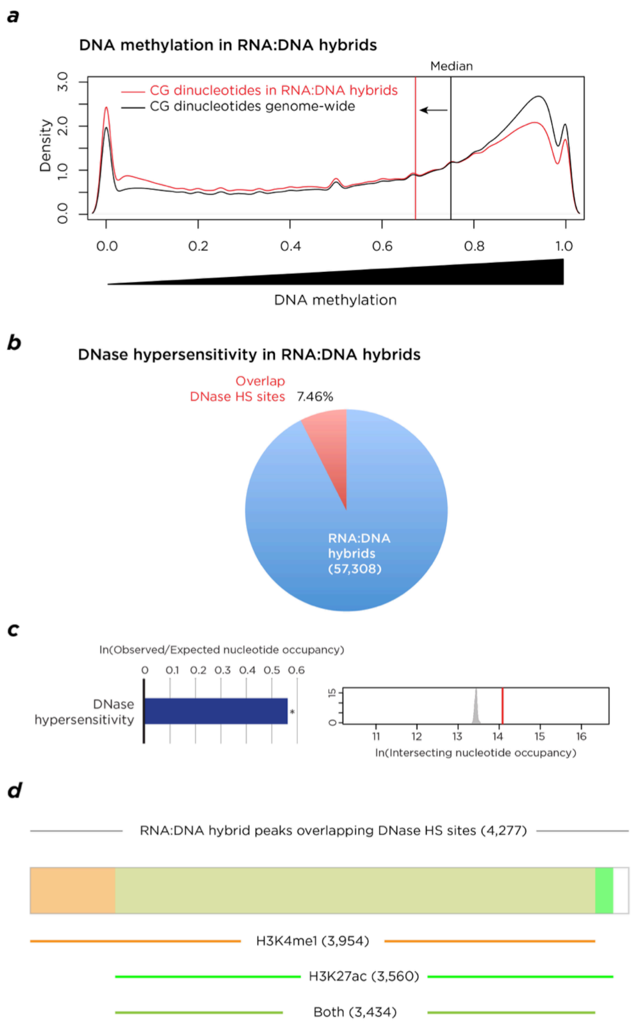

Figure S5: Relationship of RNA:DNA hybrids to DNA methylation and DNase hypersensitivity.

In (a) we show the DNA methylation distribution for CG dinucleotides located within RNA:DNA hybrids (red) compared with the genome as a whole (black). A modest shift towards decreased DNA methylation is apparent. In (b) we show the proportion of RNA:DNA hybrids that overlaps DNase hypersensitive (HS) sites is 7.46%, and that this is significantly enriched by permutation analysis (c). In (d) we show that most of the RNA:DNA hybrids overlapping DNase hypersensitive sites are also found to have H3K4me1 and H3K27ac histone modifications, indicating active enhancer function.

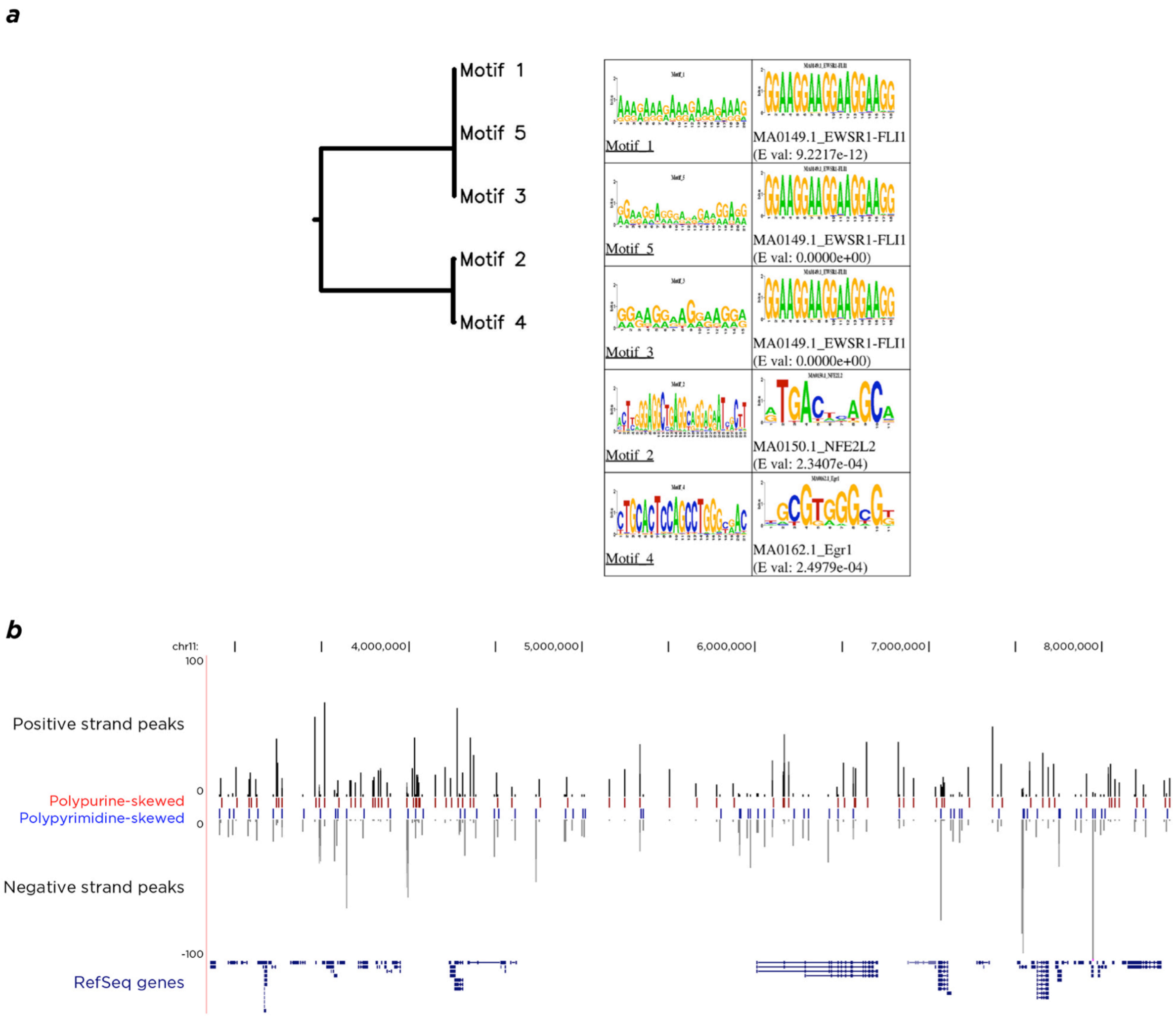

Figure S6: Motif and macro-organizational analyses.

In (a) we show the results of motif analysis of RNA:DNA hybrids, showing enrichment of the (GGAA)n motif. In (b) we show several megabasepairs of the genome that illustrates how RNA:DNA hybrids can vary in their density over a scale of hundreds of basepairs.

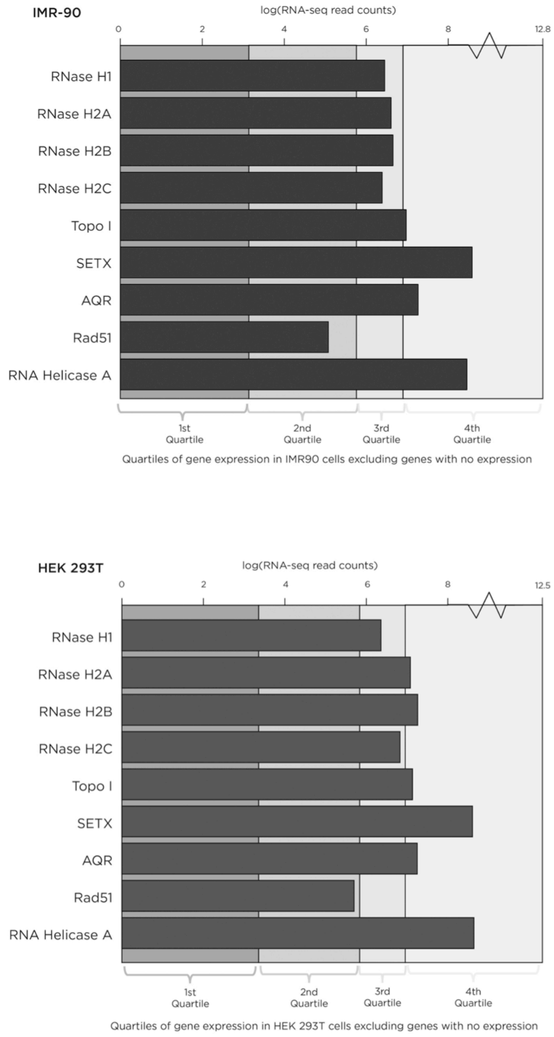

Figure S7: RNA-seq studies of genes that remove RNA:DNA hybrids.

We show the results of RNA-seq of a panel of genes known to be involved in removing RNA:DNA hybrids *in vivo*, with background shading indicating expression quartiles. Most of these genes are expressed in the two highest quartiles.

The nucleotide and chromatin characteristics and transcriptional relationships of RNA:DNA hybrids in the human genome.

Julie Nadel, Rodoniki Athanasiadou, Christophe Lemetre, N. Ari Wijetunga, Pilib Ó Broin, Hanae Sato, Zhengdong Zhang, Jeffrey Jeddeloh, Cristina Montagna, Aaron Golden, Cathal Seoighe, John M. Greally

Supplemental Experimental Procedures

Cell culture conditions

IMR-90 cells are female human lung fibroblasts (ATCC CCL-186) and HEK 293T are epithelial cells from fetal human kidney containing the SV40 T-antigen. Both cell lines were grown in Dulbecco's modified Eagle medium (DMEM) supplemented with 10% fetal bovine serum and 2mM Penicillin-Streptomycin (Invitrogen), at 37°C in 5% CO_2. The cells were allowed to reach 80-90% confluence before harvesting with trypsin-EDTA.

S9.6 antibody validation

The antibody was validated using an electrophoretic mobility shift assay (EMSA) to test the binding of the S9.6 antibody to RNA:DNA hybrid oligonucleotides. RNA:DNA hybrid oligonucleotides, and control DNA:DNA and RNA:RNA oligonucleotides, were incubated with the S9.6 antibody for 15 minutes at room temperature with a 1X EMSA buffer, and then run on a 6% native acrylamide gel. This allows visualization of the binding of the antibody through a slowed progression through the gel of the oligonucleotides.

The S9.6 antibody binding specifically to RNA:DNA hybrids was also validated through southwestern blotting. RNA:DNA, DNA:DNA, RNA:RNA, ssRNA and ssDNA oligonucleotides were blotted on a Hybond-N+ nylon transfer membrane (additionally this assay was preformed after treating the oligos with endonucleases). The membrane is then blocked overnight in 5% milk, followed by a two hour incubation with the primary S9.6 antibody. The membrane is then washed with 1% milk, incubated with the secondary antibody (anti-mouse) for one hour, washed again with 1% milk, and exposed using WestFemto Super Signal (Thermo Scientific). The oligonucleotide sequences used for this study for both the EMSA and South-Western blot are provided in **Tables S7 and S9**.

Image acquisition

FISH images were acquired with a manual inverted fluorescence microscope (Axiovert 200, Zeiss) with fine focusing oil immersion lens (x40, NA 1.3 oil). Multiple focal planes were obtained for each channel to ensure that signals on different focal planes were included. The resulting fluorescence emissions were collected using 425-475 nm (for DAPI), 546-600 nm (for spectrum orange) and 470 -497 nm (for Spectrum Green- aadUTP) filters. The microscope was equipped with a Camera Hall 100 and the Applied Spectral Imaging software.

Motif analysis

Based on the co-ordinates of RNA:DNA hybrid peaks called by the MACS algorithm (Zhang et al., 2008), 21,044 sequences were extracted using the Galaxy 'Extract Genomic DNA' tool. These sequences ranged in length from 249-31,714 bp and, in total, comprised approximately 14.1 Mb. The *de novo* motif analysis was carried out using CUDA-MEME (Liu et al., 2010), a

CUDA-enabled implementation of the MEME algorithm (Bailey and Elkan, 1994) designed to leverage the power of GP-GPU computing. The algorithm was run on an Nvidia Tesla M2050 graphics card containing 448 CUDA cores, and was configured to return only the top motif based on analysis of both forward and reverse strands using the Zero or One Occurrence Per Sequence (zoops) model, with an allowable motif width in the range 8-50 bp. The returned motif was 21 bp in length with an E-value of 9.1e-79728 and was present in 19,835 (~94%) of the extracted sequences.

References

Bailey, T.L., and Elkan, C. (1994). Fitting a mixture model by expectation maximization to discover motifs in biopolymers. Proc Int Conf Intell Syst Mol Biol *2*, 28-36.

Liu, Y., Schmidt, B., Liu, W., and Maskell, D.L. (2010). CUDA–MEME: Accelerating motif discovery in biological sequences using CUDA-enabled graphics processing units. Pattern Recognition Letters *31*, 2170-2177.

Zhang, Y., Liu, T., Meyer, C.A., Eeckhoute, J., Johnson, D.S., Bernstein, B.E., Nusbaum, C., Myers, R.M., Brown, M., Li, W., *et al.* (2008). Model-based analysis of ChIP-Seq (MACS). Genome Biol *9*, R137.

Directional Whole Transcriptome Sequencing Protocol

Reagents:

Ribo-Zero™ Gold Kits (Human/Mouse/Rat): Epicentre, Cat # MRZG126
miRNeasy Mini Kit: QIAGEN, Cat #217004
RNase-Free DNase Set (50): QIAGEN, Cat#79254
SuperScript® III First-Strand Synthesis System: Invitrogen, Cat # 18080-051
Actinomicyn D: Sigma, Cat. #A1410-2MG
dUTP set (100 µmol each): Promega, Cat. #U-1335
Second Strand Buffer: Invitrogen, Cat.# 10812-014
E. coli DNA ligase: Invitrogen, Cat.# 18052-019
E. coli DNA polymerase: Invitrogen, Cat.# 18010-025
Quick Ligation Kit: NEB, Cat. #M2200S
T4 DNA Polymerase: NEB, Cat. #M0203S
T4 DNA Polynucleotide kinase: NEB, Cat. #M0201S
T4 DNA ligase buffer: NEB, Cat.# B0202S
Klenow Fragment (3'->5' exo -): NEB, Cat. #M0212S
DNA Clean & Concentrator™-5: Zymo research, Cat#D4013 (50 prep) or D4014 (200 prep)
Phusion High-Fidelity DNA Polymerase: NEB, Cat. #F-530S
100 mM dNTP nucleotides: Invitrogen, Cat. #10297-018
UNG: Fermentas, Cat. #EN0361
Ethachinmate (replaces Glycogen): Wako USA, Cat. #312-01791
TruSeq adapters: Illumina (Epigenomic Shared Facility)
GelGreen Nucleic Acid Gel Stain (replaces SYBRGreen): Biotium, Cat. #41005, VWR international, Cat. #89139-146
MinElute Gel Extraction Kit (50): QIAGEN, Cat#28604

Note about RNase contamination:

Ribonuclease contamination is a significant concern for working with RNA. We highly recommend that you:

1) *Always use RNase-free tubes and pipette tips*
2) *Always wear gloves when handling samples containing RNA. Change gloves frequently, especially after touching potential sources of RNase contamination.*
3) *Always wear gloves when handling kit components.*
4) *Keep all kit components tightly sealed when not in use. Keep all tubes containing RNA tightly sealed during the incubation steps.*

RNA extraction

Select RNA extraction method based on your research design.

DNase Treatment

a. RNA sample (5-10µg of total RNA and adjust volume)	43µl
b. RNase OUT	1µl
c. DNase	1µl
d. 10X DNase Buffer	5µl

Note: If you have used Trizol for RNA extraction, be aware that is important to remove any guanidinium salts remaining before starting the rDNA removal. We generally use the miRNeasy Mini Kit (QIAGEN) to clean up again.

Ribo-Zero rRNA Removal

We use Ribo-Zero rRNA Removal Kit (Human/Mouse/Rat) Low Input from Epicentre.

Follow the instructions from Epicentre. We suggest to use 1 µl of Ethachinemate instead of 2 µl of Glycogen at purify the rRNA-depleted sample step. Re-suspend the rRNA depleted RNA in 5 µL of RNase-free water.

Check point: We usually run a BioAnalyzer (Agilent) chip (through our core facility) to ensure our RNA is not degraded. Measure your RNA concentration using NanoDrop.

Directional RNA-seq library preparation

1) **First Strand Synthesis**

Set up reaction in a PCR tube as follows:

a. 10X RT buffer 1 µl
b. 20 ng Random hexamers (50 ng/µl) 0.8 µl
c. 25 pmol Oligo dT (50 µM) 0.5 µl
d. 0.1M DTT 0.85 µl
e. 25 mM $MgCl_2$ 1.7 µl
f. dNTP (10 mM) 0.425 µl
g. RiboZero treated RNA 3.225 µl

Total - 8.5 µl

Incubate at 98°C for 1 minute

Ramp down (0.1°C/second) to 70°C

Incubate at 70°C for 5 minutes

Ramp down (0.1°C/second) to 15°C

Add:

h. Actinomicin D (125 ng/µl) 0.5 µl
i. RNase OUT 0.5 µl
j. SuperScript III 0.5 µl

Total – 10 µl

Incubate at 25°C for 10 minutes

Ramp up (0.1°C/second) to 42°C

Incubate at 42°C for 45 minutes

Ramp up (0.1°C/second) to 50°C

Incubate at 50°C for 25 minutes

Incubate at 75°C for 15 minutes

--Ethanol Precipitate:

1. Adjust the volume of each sample to 50 µl using RNase-Free water
2. Add 5 µl of 3 M Sodium Acetate (From RiboZero Kit) to each tube
3. Add 1 µl of Ethachinmate (replacing glycogen) to each tube and mix with gentle vortexing
4. Add 250 µl of 100% ethanol to each tube and mix thoroughly by gentle vortexing

5. Place the tubes at -20°C for at least one hour (alternative overnight to continue the rest of the protocol the next day)
6. Centrifuge the tubes at >10,000x g in a microcentrifuge for 30 minutes at 4°C. Carefully remove and discard the supernatant.
7. Wash the pellet with ice cold 70% Ethanol and centrifuge at >10000x g for 5 minutes. Carefully remove and discard the supernatant.
8. Centrifuge briefly to collect any residual supernatant, carefully remove and discard. Allow the pellet to air dry at room temperature for 5 minutes.
9. Dissolve the pellet in 10 µl of the buffer:
    a. 10x RT buffer 1 µl
    b. 0.1 M DTT 1 µl
    c. 25 mM $MgCl_2$ 2 µl
    d. RNase-free water 6 µl

    Total – 10 µl

2) Second strand Synthesis

Add 45 µl of RNase Free water to RNA sample

Set up reaction in a PCR tube as follows:

a. 5x ss buffer 15 µl
b. 10mM dUTP 2 µl
c. E coli DNA Ligase 0.5 µl
d. E coli DNA polymerase 2 µl
e. RNase H 0.5 µl
f. RNA (from Step 1) 55 µl
g. H_2O to 75 µl for reaction

Incubate for 2 hours at 16°C (easy in a thermocycler)

h. Add T4 DNA polymerase 1 µl

Incubate for the following: 16°C - 10 minutes

75°C - 20 minutes

37°C - 15 minutes

--Purify with Zymo DNA Clean and Concentrator Kit

- Elute in 22 µl

Note: We are careful to remove any extra PE after wash with 10 µl pipette tip from lid of the tube as this has been seen to drastically affect library outcomes.

Fragmentation - We usually ask our Epigenomics Shared Facility to shear samples with the Covaris, with a fragmentation range of 200-300 bp.

After fragmentation, purify with Zymo DNA Clean and Concentrator Kit. Elute in 22 μl.

3) **Repair Ends**

   a. DNA 16 μl
   b. H_2O 3.5 μl
   c. 10x buffer (T4 ligase+ATP) 2.5 μl
   d. 10 mM dNTPs 0.5 μl
   e. T4 DNA polymerase 1.25 μl
   f. T4 PNK 1.25 μl

   Total – 25 μl

   Incubate at 20°C for 30 minutes.

   Purify with Zymo DNA Clean and Concentrator Kit

   Elute in 18 μl

4) **Add A Overhangs**

a. DNA 16 µl
b. 10X NEB2 2.5 µl
c. 1mM ATP 5 µl
d. Klenow (exo-) 1.5 µl

Incubate for 30 minutes at 37°C

Purify with Zymo DNA Clean and Concentrator Kit

Elute in 12 µl

5) **Adapter ligation (TruSeq adapters)**

a. DNA (from 3) 10 µl
b. 2x ligation buffer 15 µl
c. Adapters 0.5 µl
d. Quick ligase 1.5 µl

Incubate for 15 minutes at room temperature

Purify with Zymo DNA Clean and Concentrator Kit

Elute in 20 µl

6) **UNG Treatment**

Set up the following reaction on ice:

a. 5X HF buffer 10 µl
b. 10 µM forward primer 1.75 µl
c. 10 µM reverse primer 1.75 µl
d. dNTP mix 1 µl
e. H_2O 14 µl
f. DNA 20 µl
g. UNG 1 µl

Incubate at: 37°C - 15 minutes
98°C - 10 minutes

h. Add Phusion 0.5 µl

Incubate at: 98°C - 2 minutes
18 cycles of:
----98°C – 30 seconds
----60°C – 30 seconds
----72°C – 15 seconds

72°C – 10 minutes

Hold at 4°C

Clean immediately using Zymo DNA Clean and Concentrator Kit

Elute in 12 µl (1 µl for Qubit, 1 µl for Bioanalyzer, 10 µl for sequencing)

7) **Library analysis**

We use Qubit® Fluorometric Quantitation for our final library quantitation, and visualize libraries through Bioanalyzer High Sensitivity DNA Chip through our Epigenomic Shared Facility.

Libraries should show a smear from ~200-600 bp. Check for adapter dimer. If you have adapter dimer, purify your library using gel extraction. For the gel extraction, we run the libraries in a 3% Low Range Ultra agarose stained with GelGreen, using a 20 bp molecular ladder and a blue light box (Dark Reader).

Directional RNA:DNA Hybrid Immunoprecipitation Sequencing Full Protocol

Reagents:

Proteinase K: Invitrogen, Cat. # 25530049
S9.6 Antibody-Producing Hybridoma Line: ATCC, Cat. #HB08730
RNase I: Ambion, Cat. # AM2294
M-280 Sheep anti-mouse Dynabeads: Invitrogen, Cat. #112.01D
dUTP set (100 µmol each): Promega, Cat. #U-1335
Second Strand Buffer: Invitrogen, Cat.# 10812-014
E. coli DNA ligase: Invitrogen, Cat.# 18052-019
E. coli DNA polymerase: Invitrogen, Cat.# 18010-025
Quick Ligation Kit: NEB, Cat. #M2200S
T4 DNA Polymerase: NEB, Cat. #M0203S
T4 DNA Polynucleotide kinase: NEB, Cat. #M0201S
T4 DNA ligase buffer: NEB, Cat.# B0202S
Klenow Fragment (3'->5' exo -): NEB, Cat. #M0212S
DNA Clean & Concentrator™-5: Zymo research, Cat#D4013 (50 prep) or D4014 (200 prep)
Phusion High-Fidelity DNA Polymerase: NEB, Cat. #F-530S
100 mM dNTP nucleotides: Invitrogen, Cat. #10297-018
UNG: Fermentas, Cat. #EN0361
Ethachinmate (replaces Glycogen): Wako USA, Cat. #312-01791
TruSeq adapters: Illumina (Epigenomic Shared Facility)
GelGreen Nucleic Acid Gel Stain (replaces SYBRGreen): Biotium, Cat. #41005, VWR international, Cat. #89139-146

Whole Cell Nucleic Acid (WCNA) Salting Out

1) Lyse cells with lysis buffer (2 ml/150 mm plate or flask)
    a. For HEK 293T cells we used 6 x 150 mm plates for a large stock of WCNA
2) Scrape off cells and lysis buffer into 50 ml falcon tubes, and leave rocking overnight at 37°C.
3) Add 1/3 volume saturated NaCl and shake vigorously for 15 seconds.
4) Centrifuge at 4°C for 30 minutes at 900g
    a. If only a small pellet is seen, add ¼ volume NaCl and repeat.
5) Pour supernatant into fresh tube and add 2 volumes of ice cold 100% Ethanol
    a. Remove DNA with a borosilicate glass pipette hook and wash in 70% Ethanol
    b. Briefly let air dry on hook to remove all traces of Ethanol
6) Resuspend in appropriate volume of TE (4°C for 1 hour) – adjust TE volume until all nucleic acid is resuspended
7) Phenol/chloroform extract

8) Resuspend in TE and store at -20°C.

Lysis buffer

0.5 mg proteinase K (2.5 µl of 20 mg/ml)

50 µl 20% SDS

950 µl TE-8

TE-8

0.5 M Tris pH 8

20 mM EDTA

10 mM NaCl

Immunoprecipitation Sample Preparation

1) Extract WCNA with the salting out method
2) Sonicate 1 ml of WCNA at a concentration of 200 ng/ml (using nanodrop for concentration measurement) to ~400 bp fragments
    a. We use the Covaris sonicator: 5 cycles of 10 seconds on at 10%, 1 minute off
3) Do an RNase I digestion:
    a. WCNA 100 µl (20 µg, Nanodrop)
    b. 5 M NaCl 6 µl
    c. H_2O 89 µl
    d. RNase I 5 µl

    Incubate at 37°C for 1.5 hours
4) Phenol/chloroform extract the reaction and precipitate with Ethanol. Resuspend sample with EB to 30 µl and nanodrop for concentration.

Note: Do not suspend in TE, because the EDTA in TE will terminate the RNase H reaction in the second strand synthesis of library preparation.

At this point, set aside some sonicated, RNase I treated WCNA as your input, which you will use for library preparation.

RNA:DNA Hybrid Immunoprecipitation

1) Add in this order in a 2 ml tube:
    a. 3 µg WCNA
    b. H_2O to final volume of 750 µl

c. 10x IP buffer 75 µl

d. S9.6 antibody (3 mg/ml) 20 µl

2) Incubate at 4°C with rotation overnight

3) Next day, wash the necessary amount of the M-280 Sheep anti-mouse Dynabeads with 1x IP buffer (for 60 µl per sample)

4) Add 60 µl of Dynabeads in each tube and incubate at 4°C for 2 hours with rotation

5) Isolate beads using magnet and remove supernatant

6) Wash the beads 3x with 1ml 1x IP buffer, minimizing pipetting, and changing to a new tube each time.

7) Re-suspend beads at 500µl 1x IP buffer and add 100 µg proteinase K (20 mg/ml, Invitrogen) - incubate for 2 hours at 37°C .

8) Add another 60 µg Proteinase K and continue the incubation for another 1 hour at 37°C.

9) Phenol/chloroform extract the supernatant (discarding the beads) and precipitate in the presence of 1 µl Ethachinmate (glycogen substitute).

10) Resuspend pellet in 40 µl EB.

Note: Do not suspend in TE, because the EDTA in TE will terminate the RNase H reaction in the second strand synthesis of library preparation.

<u>10x IP buffer (for 50 ml)</u>

100 mM Na-Phosphate buffer pH 7.0

1.4 M NaCl (14 ml 5M NaCl)

0.5 % Triton X-100 (250 µl)

(10x and 1x IP buffer can be kept at RT for several weeks)

Directional RDIP-seq library preparation

1) **Second strand synthesis**

Set up reaction in a PCR tube as follows:

a.	Up to 1 μg of sample	
b.	10 mM dUTP	1.5 μl
c.	5x ss buffer	15 μl
d.	BSA (NEB)	0.5 μl
e.	E coli DNA Ligase	0.5 μl
f.	E coli DNA polymerase	2 μl
g.	RNase H	0.5 μl
h.	H_2O	to 75 μl for reaction

Incubate for 2 hours at 16°C (thermocycler use recommended for this step)

i.	Add T4 DNA polymerase	1 μl

Incubate for the following: 16°C - 10 minutes
75°C - 20 minutes
37°C - 15 minutes

--Purify with Zymo DNA Clean and Concentrator Kit

- Elute in 22 μl

Note: We are careful to remove any extra PE after wash with 10 μl pipette tip from the lid of the tube as this has been seen to drastically affect library outcomes.

2) **Fix Ends**

a.	DNA from 1	20 μl
b.	10x buffer (T4 ligase +ATP)	5 μl
c.	10 mM dNTPs	2 μl
d.	T4 DNA polymerase	2.5 μl
e.	T4 PNK	2.5 μl
f.	H_2O	18 μl

Incubate for 1 hour at room temperature

Purify with Zymo DNA Clean and Concentrator Kit

Elute in 18 μl

3) **Add A Overhangs**

a.	DNA from 2	16 μl
b.	10X NEB2	2.5 μl

c. 1 mM ATP 5 µl

d. Klenow (exo-) 1.5 µl

Incubate for 30 minutes at 37°C

Purify with Zymo DNA Clean and Concentrator Kit

Elute in 12 µl

4) Adapter ligation (TruSeq adapters)

a. DNA from 3 10 µl

b. 2x ligation buffer 15 µl

c. Adapters 2.5 µl

d. Quick ligase 2.5 µl

Incubate for 15 minutes at room temperature

Purify with Zymo DNA Clean and Concentrator Kit

Elute in 20 µl

5) UNG Treatment

a. 5X HF buffer 10 µl

b. 10 µM forward primer 1.75 µl

c. 10 µM reverse primer 1.75 µl

d. dNTP mix 1 µl

e. H_2O 14 µl

f. Product from 4) 20 µl

g. UNG 1 µl

Incubate at: 37°C - 15 minutes

98°C - 10 minutes

h. Add Phusion 0.5 µl

Incubate at: 98°C - 2 minutes

18 cycles of:

----98°C – 30 seconds

----60°C – 30 seconds

----72°C – 15 seconds

72°C – 10 minutes

Hold at 4°C

Clean immediately using Zymo DNA Clean and Concentrator Kit

Elute in 12 µl (1 µl for Qubit, 1 µl for Bioanalyzer, 10 µl for sequencing)

6) **Library analysis**

We use Qubit® Fluorometric Quantitation for our final library quantitation, and visualize libraries using the Bioanalyzer High Sensitivity DNA Chip through our Epigenomic Shared Facility.

Libraries should show a smear from ~200-600 bp. Check for adapter dimer. If you have adapter dimer, purify your library using gel extraction. For the gel extraction, we run the libraries in a 3% Low Range Ultra agarose stained with GelGreen, using a 20 bp molecular ladder and a blue light box (Dark Reader).
